# *Cingulin b* Is Required for Zebrafish Lateral Line Development Through Regulation of Mitogen-Activated Protein Kinase and Cellular Senescence Signaling Pathways

**DOI:** 10.3389/fnmol.2022.844668

**Published:** 2022-05-05

**Authors:** Yitong Lu, Dongmei Tang, Zhiwei Zheng, Xin Wang, Na Zuo, Renchun Yan, Cheng Wu, Jun Ma, Chuanxi Wang, Hongfei Xu, Yingzi He, Dong Liu, Shaofeng Liu

**Affiliations:** ^1^Department of Otolaryngology-Head and Neck Surgery, Yijishan Hospital of Wannan Medical College, Wuhu, China; ^2^State Key Laboratory of Medical Neurobiology and MOE Frontiers Center for Brain Science, ENT Institute and Department of Otorhinolaryngology, Eye and ENT Hospital, Fudan University, Shanghai, China; ^3^NHC Key Laboratory of Hearing Medicine, Fudan University, Shanghai, China; ^4^Nantong Laboratory of Development and Diseases, School of Life Sciences, Co-innovation Center of Neuroregeneration, Key Laboratory of Neuroregeneration of Jiangsu and MOE, Nantong University, Nantong, China; ^5^Department of Forensic Medicine, Soochow University, Suzhou, China

**Keywords:** *cingulin b*, zebrafish, development, MAPK signaling pathway, cellular senescence

## Abstract

Cingulin, a cytoplasmic element of tight junctions (TJs), is involved in maintenance of the integrity of epithelial and endothelial cells. However, the role of cingulin in the development of auditory organs remains unclear. Zebrafish is popular as a model organism for hearing research. Using the whole mount *in situ* hybridization (WISH) experiment, we detected the expression of *cingulin b* in the posterior lateral line system (PLLs) of zebrafish. We traced the early development progress of zebrafish PLLs from 36 hpf to 72 hpf, and found that inhibition of *cingulin b* by target morpholinos resulted in severe developmental obstruction, including decreased number of neuromasts, reduced proliferative cells in the primordium, and repressed hair cell differentiation in the neuromasts. To examine the potential mechanism of *cingulin b* in the development of zebrafish PLL neuromasts, we performed RNA-seq analysis to compare the differently expressed genes (DEGs) between *cingulin b* knockdown samples and the controls. The KEGG enrichment analysis revealed that MAPK signaling pathway and cellular senescence were the key pathways with most DEGs in *cingulin b*-MO morphants compared to the Control-MO embryos. Furthermore, quantitative RT-PCR analysis confirmed the findings by RNA-seq that the transcript levels of cell cycle negative regulators such as *tp53* and *cdkn1a*, were remarkably upregulated after inhibition of *cingulin b*. Our results therefore indicated an important role of *cingulin b* in the development of auditory organs, and MAPK signaling pathway was inhibited while cellular senescence pathway was activated after downregulation of *cingulin b*. We bring forward new insights of cingulin by exploring its function in auditory system.

## Introduction

Tight junctions (TJs), mainly composed of claudins, occludin, ZO proteins, cingulin and paracingulin, are widely localized at the apicolateral borders of cells, and play important roles in maintaining the integrity, permeability and polarity of cells (González-Mariscal et al., 2014; Citi, 2019). Cingulin is localized in the cytoplasmic region of TJs, comprised of a head, a rod and a tail domain (Cordenonsi et al., 1999). Cingulin connects to actin and microtubule cytoskeleton in the head domain, and interacts with Rho family GTPases in the coiled-coil rod region (Cordenonsi et al., 1999; D’atri et al., 2002; Ohnishi et al., 2004; Van Itallie et al., 2009; Yano et al., 2013). Cingulin is mainly involved in regulating the paracellular and blood-brain barrier, for example, edema is more severe in the specific cingulin knock-out mouse model compared to the controls (Hawkins and Davis, 2005; Zhuravleva et al., 2020). In addition, cingulin is found expressed in the organ of Corti, and its distribution is rearranged after high-intensity noise exposure (Raphael and Altschuler, 1991). In a kanamycin damaged guinea pig model, cingulin together with adherens junctions such as E-cadherin and beta-catenin are found reorganized in two distinct planes, and they would preserve the integrity of tissues during scar formation and hair cell degeneration, indicating a barrier function of cingulin in the organ of Corti (Leonova and Raphael, 1997). Cingulin is also expressed in key regions of mouse cochlea, such as spiral ligament, stria vascularis, spiral limbus, and tectorial membrane (Batissoco et al., 2018). However, the role of cingulin in the development of auditory system is unknown.

Zebrafish have a high genetic similarity with the genome of human, and many critical genes required for the development of eyes, ear, brain, heart and other organs are highly conserved between zebrafish and humans, which makes zebrafish an excellent model for studying the human disease (Dooley and Zon, 2000; Howe et al., 2013; Kalueff et al., 2014). Besides, the characteristics of short reproductive cycle, strong reproductive ability, and transparent embryos increase the popularity of zebrafish as an animal model compared to mice (Mandrekar and Thakur, 2009; He et al., 2017). The mature neuromast of zebrafish PLL is consisted of the central hair cells (HCs) and the surrounding supporting cells (SCs), which share many structural and functional similarities with the inner ear cochlea of mammals (Nicolson, 2005), making zebrafish lateral line system a significant model for studying hair cell development, survival and regeneration (Driever et al., 1994; Pyati et al., 2007; Brignull et al., 2009).

In this study, we chose zebrafish as the animal model to explore the potential role of cingulin in the development of lateral line system of zebrafish. In zebrafish, *cingulin b* is orthologous to human cingulin. We firstly designed anti-sense morpholinos to downregulate the expression of *cingulin b*, and the efficacy of *cingulin b*-MO was confirmed by ISH staining and qPCR analysis of *cingulin b*. We observed reduced number of neuromasts, decreased cell proliferation, and repressed HC differentiation in the PLL system of zebrafish after knocking down *cingulin b* compared to the control group. The RNA-seq analysis revealed that MAPK signaling pathway and cellular senescence genes were involved in the development of zebrafish PLL after inhibition of *cingulin b*. Our findings uncover a potential role of cingulin in the development of zebrafish mechanosensory organs.

## Materials and Methods

### Animal Operations

All zebrafish, including the wild type AB line and the transgenic *Tg (cldnb: lynGFP)* and *Tg (brn3c: mGFP)^s^*^356*t*^ lines were bred in 28.5°C constant temperature incubator in embryo medium according to the standard formula (Kimmel et al., 1995). The stage of embryonic development was marked as hours- or days- after fertilization (hpf or dpf) (Kimmel et al., 1995). In order to avoid pigmentation, the embryos should be further immersed in 1-phenyl-2-thiourea (PTU) (Sigma-Aldrich) in the culture medium from 10 hpf (Tang et al., 2019). The operations on zebrafish were discussed and permitted by the Animal Conservation and Utilization Committee of Fudan University in Shanghai.

### Morpholino Injection and mRNA Rescue Test

*Cingulin b*-MO, sequenced in 5’-TCCTGTCCGCAGAGAGGG AACTCAT-3’, was injected at a dose of 2 ng or 3 ng at one or two cell stage of embryos to reduce the expression of *cingulin b*. The other siblings were considered as controls by injection with a sequence of 5’-CCTCTTACCTCAGT TACAATTTATA-3’, namely control-MO (Control-MO). For the messenger RNA (mRNA) rescue experiment, a mixture of *cingulin b*-MO and *cingulin b* mRNA (Forward primer: 5’-AT GAGTTCCCTCTCTGCGGA-3’; Reverse primer: 5’-TCAACAG CTGGTGGTCTGAA-3’) was injected at the same stage with other groups.

### Whole Mount *in situ* Hybridization in Zebrafish

WISH experiment was operated as previously disclosed (He et al., 2014; Thisse and Thisse, 2014). To examine the expression pattern of *cingulin b* in zebrafish, we collected embryos at various stages including 3.7, 14, and 48 hpf. To verify the efficacy of *cingulin b*-MO in the lateral line system of zebrafish, we collected embryos at 48 hpf. After a series of gradient solutions for dehydration, the collected embryos were stored in pure methanol (100% concentration) at −20°C. Before hybridization, the embryos would be gradient rehydrated first, and then digested with 20 μg/ml protease K. The probe was added and hybridized at 65°C constant temperature overnight. After thorough washes with the SSC-series at 65°C, the embryos were blocked in 2x BBR at room temperature for at least 1 h. Anti- digoxigenin (Dig)-AP Fab fragment (Roche) was added and incubated with specimens overnight at 4°C. Primers for synthesizing the objective genes were listed in Supplementary Table 1. Color reaction was implemented with BM purple AP substrate (Roche) in the dark at 37°C, and stopped with NTMT. The embryos after three times rinses were then re-fixed in 4% PFA and treated with different gradients of glycerol/PBS. The final specimens were stored in 100% glycerol and photographed by fluorescence stereoscopic microscope. All images were prepared by Photoshop and Illustrator software (2018, Adobe).

### BrdU Labeled Cell Proliferation Analysis and Immunohistochemical Staining

Bromodeoxyuridine (BrdU) co-incubation was conducted to label the proliferative cells. The dechorionated embryos at 34 hpf were incubated in 10 mM BrdU (Sigma-Aldrich) for 2 hours to show the cell proliferation in the PLL primordium, while the dechorionated larvae at 2 dpf were incubated in 10 mM BrdU (Sigma-Aldrich) for 24 hours to examine the proliferative cells in PLL neuromasts of zebrafish. The corresponding embryos or larvae were collected, anesthetized in 0.02%MS-222 (Sigma-Aldrich), and fixed in 4% PFA at 4°C overnight. After washing with PBT-2 for 3 times, the collected embryos were soaked in 2 N HCl at 37°C for 30 min. After incubation with the primary anti-BrdU monoclonal antibody (1:200 dilution; Santa Cruz Biotechnology) for 1 h at 37°C following 4°C overnight, the samples were washed for several times and then incubated with the secondary Cy3 polyclonal antibody (1:300 dilution; Jackson) for 1 h at 37°C. DAPI (1:800 dilution; Invitrogen) was added and incubated with the embryos or larvae for 20 min at room temperature to label the nuclei. The fluorescence-labeled embryos were imaged by Leica confocal fluorescence microscope (TCS SP8; Leica). The images obtained were further rotated, cut, and adjusted in the brightness by Photoshop (2018, Adobe) and then the images were aligned and added with fonts or labels by Illustrator software (2018, Adobe).

### RNA-Sequencing Analysis

Before specimen collection, the zebrafish embryos at 48 hpf accepted depletion of chorion and the yolk sac. The total RNA was extracted with TRIzol reagent (Thermo Fisher Science) and reversely transcribed into cDNA using the first strand of transcriptional cDNA synthesis kit (Roche). An Illumina HiSeq X Ten platform was used for library sequencing. Raw reads were firstly filtered out the data in low-quality, and the remaining high quality raw data were used for downstream analyses. We used the Spliced Transcripts Alignment to a Reference (STAR) software as the reference genome library. Differential expression analysis was conducted with the DESeq (2012) R package, and *p*-value <0.05 indicated significant difference. R package was performed for KEGG pathway enrichment analysis of DEGs on the basis of hypergeometric distribution. KEGG pathway database were the reference for further functional and pathway enrichment analysis.

### Quantitative Real-Time PCR

In order to fully quantity the mRNA level of target genes, a quantitative real-time PCR (qRT-PCR) system (LightCycler^®^480) was operated on 48 hpf larvae in the Control-MO group and the *cingulin b*-MO group, using the PrimeScript RT reagent Kit (RR047A, Takara Biomedical Technology) and the SYBR PreMix Ex Taq Kit (RR820A, Takara Biomedical Technology). ^ΔΔ^Ct method was chosen for results analysis. The primer sequences used in the study were described in Supplementary Table 2. Each qPCR assay was repeated in triplicate, and GAPDH was used as the internal reference genes.

### Statistical Analysis

All statistics were performed with GraphPad Prism software (version, 8.0c). Comparison between two groups was conducted with double-tailed Student t test, while comparisons among multiple groups were carried out by One-way ANOVA. Statistics were recorded as mean ± SEM (standard error of mean), and the difference was considered to be of significant difference with *p*-value less than 0.05.

## Results

### Expression of *Cingulin b* in Zebrafish

**FIGURE 1:**
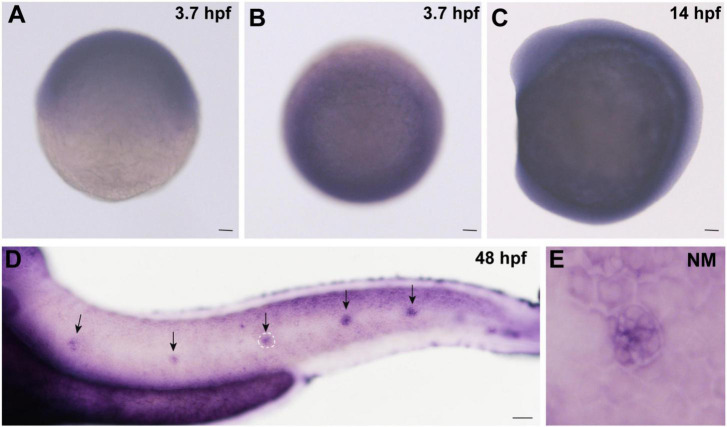
Expression of *cingulin b* is detected during the early development of zebrafish. **(A,B)** *In situ* hybridization staining of *cingulin b* at 3.7 hpf (*n* = 13) from the lateral view **(A)** and the top view **(B)**. **(C)** *Cingulin b* is expressed in the whole somite at 14 hpf from the lateral view (*n* = 14). **(D–E)** The expression of *cingulin b* is focused on the neuromasts of the posterior lateral line system at 48 hpf (*n* = 11). Scale bars mark 50 μm in panel **(A–D)**. The black arrows in D indicate neuromasts, and the white dotted lines labeled neuromast in D is magnified in panel **(E)**.

In order to detect whether *cingulin b* is expressed in zebrafish, we collected embryos at various stages and conducted WISH analysis for *cingulin b* staining. As shown in Figure 1, *cingulin b* was detected expressed in the oblong stage at 3.7 hpf (Figures 1A,B), the 10-somite stage at 14 hpf (Figure 1C), and the deposited PLL neuromasts at 48 hpf (Figure 1D), mainly in the central HC area (Figure 1E). To confirm the expression of *cingulin b* in the early development of zebrafish, we also conducted the sense control probe for *cingulin b* at 48 hpf, however, we didn’t detect any expression of *cingulin b* in the lateral line system of zebrafish compared to that using antisense mRNA probe for *cingulin b* (Supplementary Figure 1).

### *Cingulin b* Is Required for Normal Deposition of Neuromasts in Posterior Lateral Line System of Zebrafish

**FIGURE 2:**
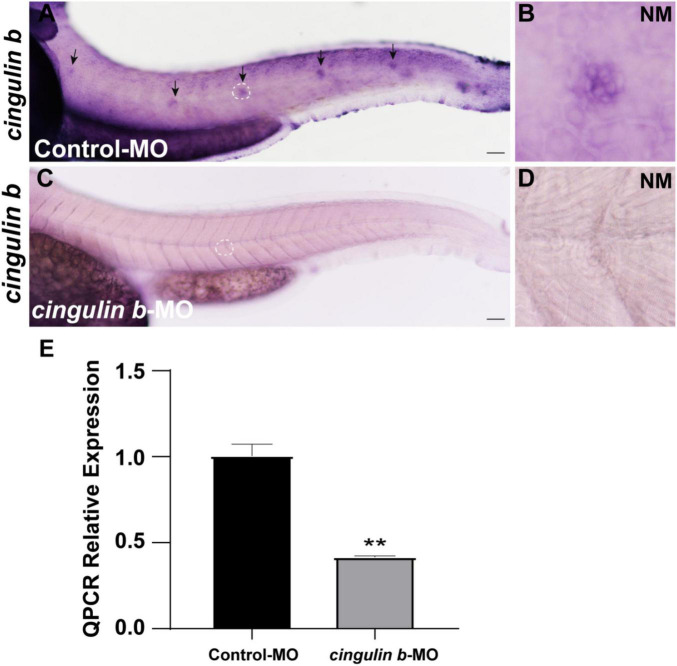
The efficacy of *cingulin b*-MO. **(A–D)** The expression of *cingulin b* is significantly reduced in the *cingulin b*-MO morphants **(C)**, *n* = 8 compared to that in the Control-MO embryos **(A)** *n* = 5. **(E)** Quantitative analysis on the level of *cingulin b* between Control-MO and *cingulin b*-MO groups (*n* = 8 in each group). Data are shown in mean ± SEM, **p < 0.01. Scale bars in panel **(A,C)** mark 50 μm. The black arrows in panel **(A)** indicate the neuromasts, and the white dotted lines labeled neuromasts in panel **(A,C)** are magnified in panel **(B,D)**, respectively.

To explore the role of *cingulin b* in the development of zebrafish, we injected specific morpholino (MO) targeting *cingulin b* at one or two cell stage of embryos for knockdown of *cingulin b*. The control group was injected with Control-MO to eliminate the effect of injection operation. The efficacy of *cingulin b*-MO-injection was examined by *in situ* staining of *cingulin b* and qRT-PCR analysis, that we found significantly down-regulated expression of *cingulin b* in the PLL neuromasts in the *cingulin b*-MO morphants compared to the controls (Figures 2A–D), and the quantitative level of *cingulin b* was remarkably decreased after *cingulin b*-MO injection compared to the embryos injected with Control-MO (Figure 2E). We also examined the embryos as a whole in the Control-MO and *cingulin b*-MO groups, and we did not find any obvious malformation in the entire zebrafish after injection with *cingulin b*-MO (Supplementary Figure 2).

**FIGURE 3:**
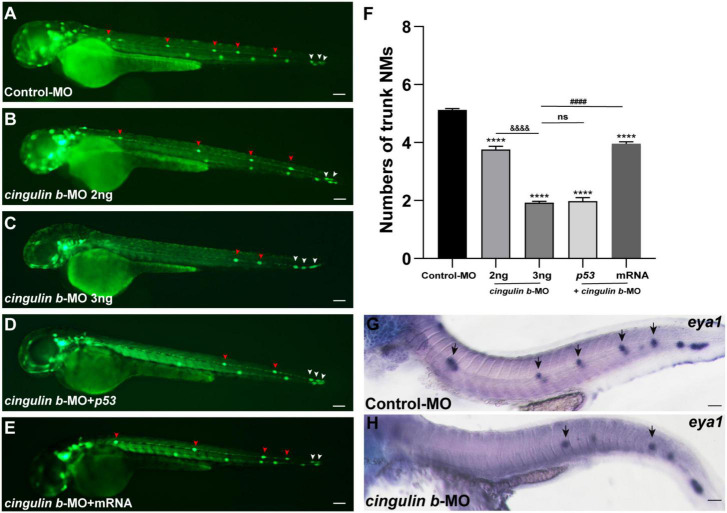
Inhibition of *cingulin b* affects the normal deposition of neuromasts in zebrafish. **(A–E)** In transgenic *cldnb:lynGFP* embryos, the neuromasts of PLL are labeled with green fluorescence. At 48 hpf, the deposition of neuromasts in zebrafish is shown in the control group **(A)**, *cingulin b* knockdown group **(B,C)**, *cingulin b*-MO + *p53* group **(D)**, and *cingulin b*-MO + *cingulin b* mRNA group **(E)**, respectively. **(F)** The number of PLL neuromasts in controls (*n* = 235), *cingulin b* knockdown (2 ng or 3 ng) group (*n* = 86 and 264, respectively), *cingulin b*-MO (3 ng) + *p53* group (*n* = 81), and *cingulin b*-MO (3 ng) + mRNA embryos (*n* = 180) at 48 hpf. The number of neuromasts decreased dose-dependently after knockdown of *cingulin b* **(A–C,F)**. The decrease in the number of neuromasts is also confirmed when co-injecting with *cingulin b*-MO and *p53* **(C,D,F)**. Combined injection of *cingulin b*-MO and *cingulin b* mRNA can partially rescue the decrease in the number of neuromasts caused by *cingulin b*-MO **(E,F)**. Red arrowheads mark the neuromasts in the trunk, and white arrowheads mark the terminal neuromasts of the PLL system **(A–E)**. Scale bars represent 100 μm. Data are shown in mean ± SEM. *Stands by the comparison with the control group: ***p < 0.0001. ^#^Stands by the comparison between *cingulin b*-MO group and *cingulin b*-MO + *cingulin b* mRNA group: ^####^*p* < 0.0001. ^&^Stands by the comparison between 2 ng *cingulin b*-MO group and 3 ng *cingulin b*-MO group: ^&⁣&⁣&⁣&^*p* < 0.0001. ns means no significance. **(G,H)** The number of *eya1* labeled neuromasts is markedly reduced after knocking down of *cingulin b* **(G)** *n* = 21 compared to the Control-MOs **(H)** *n* = 14. The black arrows in G and H indicate the neuromasts. Scale bars in panel **(G,H)** mark 50 μm.

The *Tg (cldnb: lynGFP)* zebrafish were used in this study to directly observe the morphology of neuromasts (Figure 3A). We counted the number of neuromasts at 48 hpf, a time point when the PLL primordium stops migration and finishes deposition (Kimmel et al., 1995; Nechiporuk and Raible, 2008). The average number of neuromasts in the trunk was notably decreased in the *cingulin b*-MO-injected morphants compared to that in the Control-MO-injected embryos (Figures 3A–C,F). The average number of neuromasts was even lower in 3 ng *cingulin b*-MO group than that in 2 ng *cingulin b*-MO group (Figures 3B–C,F), showing a dose-dependent manner, thus, we chose 3 ng dose for the following experiments. To avoid the non-specific effect of morpholino technology, we co-injected *p53* with *cingulin b*-MO, and surprisingly the average number of neuromasts in the trunk in *cingulin b*-MO + *p53* group was equivalent to that in *cingulin b*-MO-only group (Figures 3C–D,F). In addition, we also carried out rescue experiment, that combined injection with *cingulin b* mRNA and morpholino could partially restore the reduced number of neuromasts in the trunk (Figures 3E,F). The findings suggested that loss of *cingulin b* would affect the normal deposition of PLL neuromasts during the embryonic development of zebrafish. These findings were further validated by the expression of *eya1*, a marker for the neuromast in the lateral line of zebrafish (Kozlowski et al., 2005), that the number of neuromasts in the trunk was severely reduced in the *cingulin b*-MO morphants compared to that in the Control-MO embryos (Figures 3G,H). Taken together, our findings indicated that *cingulin b* was required in the lateral line system of zebrafish.

### Knockdown of *Cingulin b* Inhibits Cell Proliferation and Hair Cell Differentiation in the Lateral Line System of Zebrafish

**FIGURE 4:**
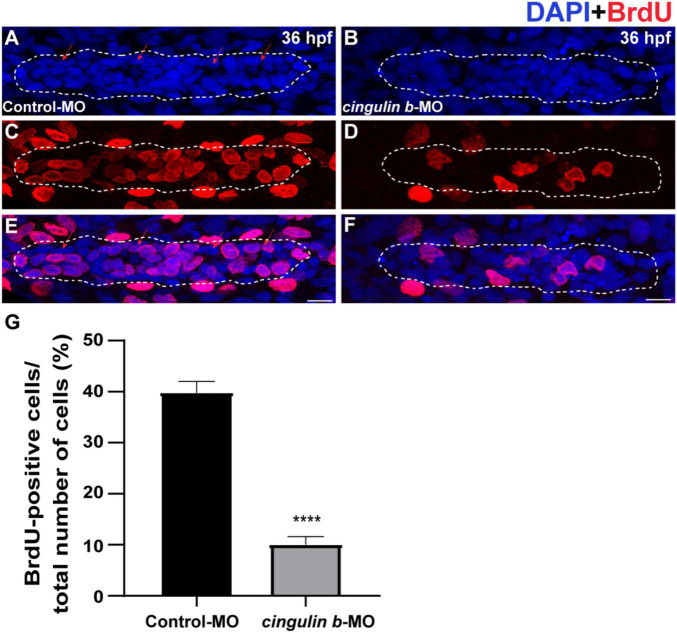
The proliferative cells in the PLL primordium are severely decreased while downregulation of *cingulin b*. **(A–F)** Representative images of BrdU positive proliferating cells and DAPI labeled nuclei in the controls **(A,C,E)** and *cingulin b*-deficient embryos **(B,D,F)** at 36 hpf. Red arrows indicate the rosette-shaped cell clusters in the primordium **(A)**. Scale bars mark the 10 μm scale. **(G)** The quantitative analysis of BrdU index in control (*n* = 16) and *cingulin b*-MO embryos (*n* = 18). Data are shown in mean ± SEM. ****p < 0.0001.

During the development of zebrafish lateral line, the collective cells migrate and form rosette-like structure in the trailing region (Aman and Piotrowski, 2008). The deposition of neuromasts occurs after assembly of the last rosette (Nechiporuk and Raible, 2008). Here, we found that cell proliferation in the primordium was destroyed during the embryonic development of zebrafish by BrdU staining after knocking down the gene expression of *cingulin b* (Figures 4A–F). BrdU index was defined as the number of BrdU-positive cells divided by the number of total cells labeled by DAPI in this article, which was used to evaluate cell proliferation ability. In 36 dpf, the BrdU index in the primordium of *cingulin b*-MO morphants decreased significantly, that the BrdU index was 10.04% ± 0.02 (*n* = 17) compared to 39.74% ± 0.02 in the Control-MO group (Figure 4G).

**FIGURE 5:**
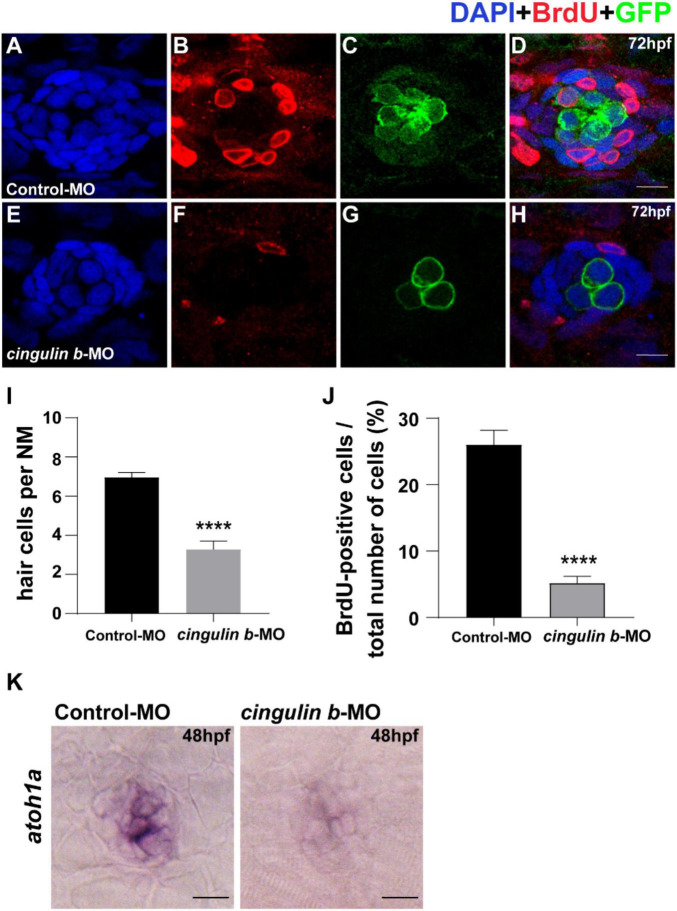
Knockdown of *cingulin b* reduces the number of HCs and cell proliferation in the neuromasts of zebrafish at 72 hpf. **(A–H)** The immunochemical staining of PLL neuromasts in the Control-MO group (*n* = 19) and *cingulin b*-MO group (*n* = 15). DAPI (green) labels nuclei **(A,E)** and BrdU (red) labels proliferative cells in the neuromast **(B,F)**. In transgenic *Tg (brn3c: mGFP)^s^*^356*t*^ lines, the membrane of HCs in PLL neuromasts are labeled with green fluorescence (GFP) **(C,G)**. **(I)** The average number of hair cells per neuromast is significantly reduced after inhibition of *cingulin b*. **(J)** BrdU index in the neuromasts is also severely downregulated in the *cingulin b*-MO injected embryos compared to the controls. Scale bars mark 10 μm **(A–H)**. Data are shown in mean ± SEM, and ****p < 0.0001. **(K)** The differentiation of HCs indicated by *atoh1* ISH staining is inhibited after injection with *cingulin b*-MO. Scale bars in panel **(K)** mark 30 μm.

To investigate the sustained effect of *cingulin b* in zebrafish embryonic development, we stained the proliferative cells with BrdU (Figures 5B,F) and collected embryos at 3 dpf. The *Tg (brn3c: mGFP)^s^*^356*t*^ zebrafish were used here because of the HCs in neuromasts were labeled with GFP (Figures 5C,G). The total cells in neuromast were labeled with DAPI (Figures 5A,E). The merged images were shown in Figures 5D,H. The number of neuromast HCs in the trunk in *cingulin b*-MO experimental group decreased significantly compared to that of Control-MO group (Figure 5I). The BrdU index was also decreased severely in the *cingulin b*-MO group compared to that in the Control-MO group (Figure 5J). We also performed ISH staining of *atohla*, a maeker of HC, and found the expression of *atoh1a* was significantly decressed after knocking down of *cigulin b* compared to the control group (Figure 5K). Altogether, the data showed that knocking down *cingulin b* inhibited cell proliferation during primordia migration and neuromasts deposition in the early development process of zebrafish PLL system.

### Mitogen-Activated Protein Kinase and Cellular Senescence Signaling Pathway Are Significantly Affected After Inhibition of *Cingulin b*

**FIGURE 6:**
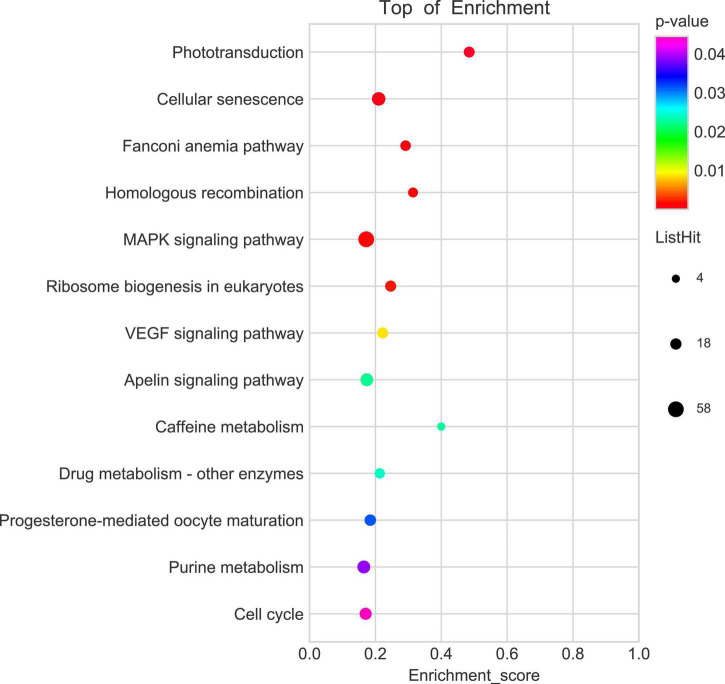
KEGG enrichment analysis screens out top 13 pathways which are highly differentiated expressed between controls and *cingulin b*-MO mutants. The analysis is conducted from three independent experiments in different groups (*n* = 30 embryos in each group), and *p* value <0.05 is considered as remarkable difference.

**FIGURE 7:**
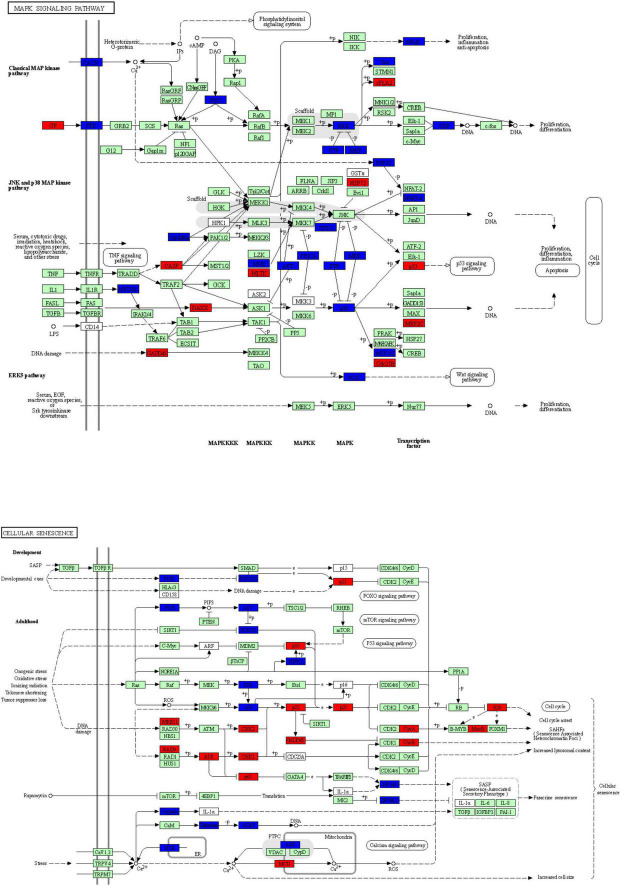
The key KEGG pathways: MAPK signaling pathway and cellular senescence signaling pathway. The red nodes represent upregulated DEGs in *cingulin b*-MO mutants, the green marked node represents downregulated DEGs in *cingulin b*-MO mutants, and the blue marked node represents overlapping targets between Control-MO and *cingulin b*-MO embryos. The analysis is conducted from three independent experiments in different groups, and each group has 30 embryos.

To explore the potential mechanism of *cingulin b* in regulating the development of zebrafish PLL system, we conducted RNA sequencing analysis to compare the difference between the control group and the *cingulin b*-MO mutants. KEGG analysis figured out the 13 top enriched pathways, of which MAPK signaling pathway and cellular senescence were the most two significant pathways evaluated by *p* value and gene counts (Figure 6). The key KEGG pathways, namely MAPK signaling pathway and cellular senescence pathway were listed in Figure 7. Also, the location of DEGs in *cingulin b*-MO siblings and overlapping genes of enriched pathways were revealed.

**FIGURE 8:**
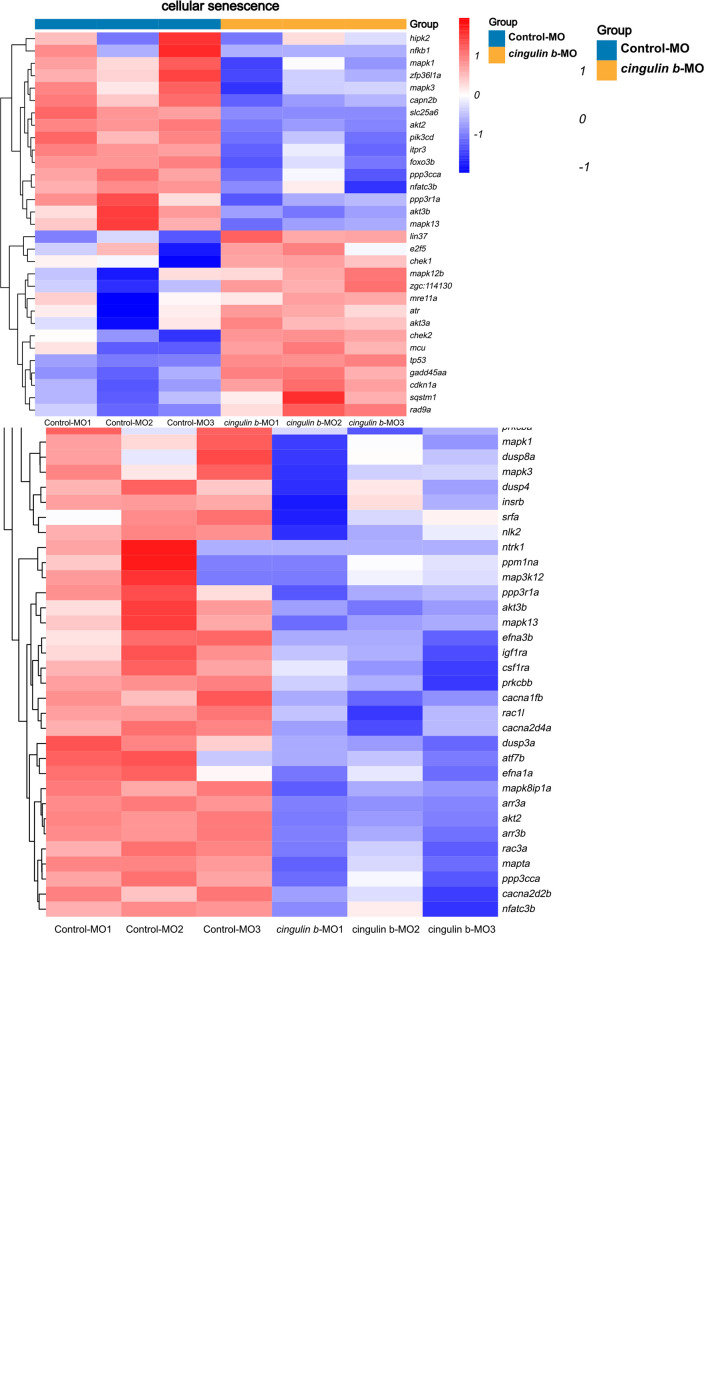
Heatmap analysis of MAPK signaling pathway and cellular senescence signaling pathway in comparison between Control-MO embryos and *cingulin b*-MO morphants. The red indicates upregulated DEGs and the blue indicates downregulated DEGs. The analysis is conducted from three independent experiments in different groups, and each group has 30 embryos.

**FIGURE 9:**
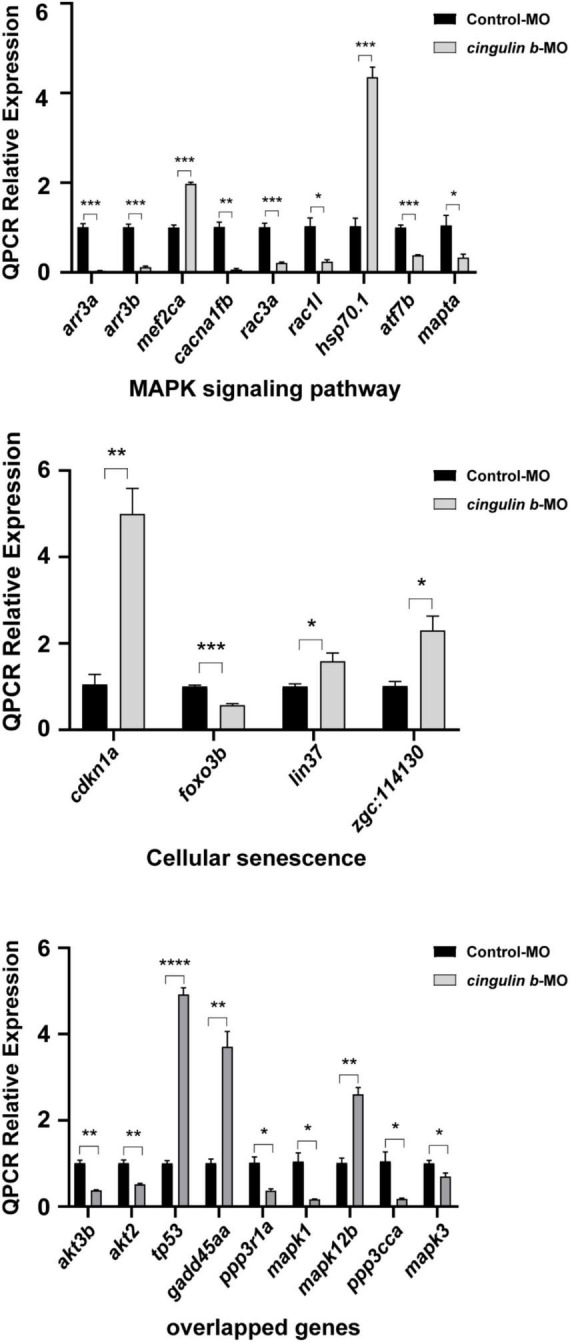
The relative mRNA levels of the indicated genes from MAPK and cellular senescence signaling pathways were normalized to the GAPDH level as determined by qRT-PCR. The results are recorded as mean ± SEM from three independent experiments (*n* = 8 embryos in each group). *p < 0.05, **p < 0.01, ***p < 0.001, and ****p < 0.0001.

Heatmap analysis of DEGs of MAPK pathway and cellular senescence was screened in Figure 8 for Control-MO group vs. *cingulin b*-MO experimental group, respectively. RT-PCR analysis for some genes from MAPK and cellular senescence signaling pathways was conducted to verify our findings in RNA-sequencing data. The primer sequences were as listed in Supplementary Table 2. As shown in Figure 9, a total 9 genes in MAPK signaling pathways, 4 genes in cellular senescence pathway, and 9 genes overlapped in the two signaling pathways were examined. The mRNA levels of *mapk1*, *mapk3*, *akt2*, *akt3b*, *atf7b*, *ppp3cca*, and *ppp3r1a* were significantly decreased after knockdown of *cingulin b*, while the expression levels of *tp53*, *mef2ca*, *mapk12b*, and *gadd45aa* were significantly increased in *cingulin b*-MO group. The results of qRT-PCR were in consistency with those found in KEGG analysis, indicating that MAPK signaling was inhibited whereas cellular senescence was activated by repression of *cingulin b*.

## Discussion

Cingulin is found interacting with connexin-26, a GJB2 encoding gene pivotal in hearing (Kelsell et al., 1997; Najmabadi et al., 2002), through the protein-protein interaction analysis (Batissoco et al., 2018). Besides, cingulin and connexin-26 are also found co-immuno-precipitated in the mouse organ of Cotri and stria vascularis (Batissoco et al., 2018). However, the role of cingulin in the cochlear development has not been identified. Previous studies have demonstrated that the PLL system of zebrafish is a good animal model for the research of mechanosensory organ development for the reason that the HCs in PLL neuromasts share similar structure and function with the mammalian inner ear HCs (He et al., 2017; Tang et al., 2019; Tang et al., 2021). In this study, we detected obvious expression of *cingulin b* in the PLL neuromasts of zebrafish. However, the number of PLL neuromasts was significantly decreased after knockdown of *cingulin b* by antisense MO injection, and we also found severe repression of cell proliferation and hair cell differentiation in the PLL primordium and neuromasts. Additionally, the RNA sequence analysis revealed that MAPK signaling was downregulated while cellular senescence signaling was upregulated in the *cingulin b*-MO embryos compared to the Control-MO injection embryos. Furthermore, we also confirmed the findings by heatmap differential analysis through qRT-PCR experiment. Our findings demonstrated that *cingulin b* was required for the normal development of zebrafish posterior lateral line by regulating the MAPK and cellular senescence signaling pathways.

Mitogen-activated protein kinase (MAPK) has been reported to be related to the formation of primordium in the posterior lateral line system of zebrafish (Harding and Nechiporuk, 2012). MAPK signaling pathway has three subfamilies, namely classical ERK pathway, Jun N-terminal kinase (JNK) pathway, and p38 pathway (Zhang and Liu, 2002). Activation of *ERK1/2* enhances cell proliferation (Lavoie et al., 2020), induces the expression of Cyclin D1 (Chen et al., 2020), and regulates the G1/S progression of cell cycle (Jirmanova et al., 2002). JNK and p38 pathways are often activated by stresses from environment or toxic agents, and usually exert antagonistic effects on cell proliferation and cell survival (Wagner and Nebreda, 2009). As previously reported, *p38* is considered as a negative regulator of cell cycle procession through downregulating cyclins and upregulating inhibitors of cyclin-dependent kinase (CDKIs) (Thornton and Rincon, 2009). In our previous study, we find that JNK inhibitor SP600125 suppresses the development of zebrafish lateral line by induction of *p21* and *p53* (Cai et al., 2016), which links the JNK pathway with tumor suppressor *p53*. Another study also demonstrates that JNK is the negative modulation of *p53* (Das et al., 2007). In the present study, *mapk1* and *mapk3* were significantly downregulated while *tp53* and *gadd45aa* were remarkably upregulated after knockdown of *cingulin b* in comparison with the Control-MO-injected controls, which were in consistency with the previous reporters.

Cellular senescence is a permanent cell cycle arrest after different damages, such as aging, oncogenes, oxidative agents, chemotherapeutic drugs, or epigenetic modulators (Hernandez-Segura et al., 2018). Senescent cells have variable phenotypes but share some common hallmarks in the mechanism, of which CDKIs are widely involved in the progression of cellular senescence, and the main components driving cell cycle arrest in senescence are *cdkn1a* (*p21*), *cdkn2a* (*p16*), and *cdkn2b* (*p15*) (Hernandez-Segura et al., 2018). Cellular senescence is found relevant to the development and tissue regeneration of zebrafish (Da Silva-Alvarez et al., 2020a,b). In this study, we observed strong elevation in the expression of *tp53*, *cdkn1a*, and *gadd45aa* in the morphants injected with *cingulin b*-MO compared to the control embryos, suggesting the activation of cellular senescence after inhibition of *cingulin b*.

## Conclusion

We demonstrate that *cingulin b* is required in the development of zebrafish lateral line system, and MAPK signaling pathway and cellular senescence are regulated by morpholino knockdown of *cingulin b*. To our knowledge, it’s the first time that the function of *cingulin b* is explored in the mechanosensory organs of zebrafish, but further studies are needed to detect direct evidence between auditory organ development and cingulin, the proteins of tight junctions.

## Data Availability Statement

The datasets presented in this study can be found in online repositories. The names of the repository/repositories and accession number(s) can be found below: https://www.ncbi.nlm.nih.gov/search/all/?term=PRJNA802059.

## Ethics Statement

The animal study was reviewed and approved by the Animal Conservation and Utilization Committee of Fudan University in Shanghai.

## Author Contributions

YH, DL, and SL: conceptualization, methodology, writing—review and editing, and project administration. YL, DT, ZZ, XW, NZ, RY, CeW, HX, JM, and CuW: methodology and formal analysis. YL, DT, and ZZ: validation, investigation, and formal analysis. All authors read and approved the final manuscript.

## Conflict of Interest

The authors declare that the research was conducted in the absence of any commercial or financial relationships that could be construed as a potential conflict of interest. The reviewer ZS declared a shared affiliation with several of the authors DT and ZZ to the handling editor at the time of the review.

## Publisher’s Note

All claims expressed in this article are solely those of the authors and do not necessarily represent those of their affiliated organizations, or those of the publisher, the editors and the reviewers. Any product that may be evaluated in this article, or claim that may be made by its manufacturer, is not guaranteed or endorsed by the publisher.

## Supplementary Material

Supplementary Figure 1 The representative S (sense control) and AS (antisense mRNA probe) images of *cingulin b* in the PLL of zebrafish at 48 hpf. Black arrowheads indicate neuromasts. Scale bars represent 50 μm, *n* = 10 in S group, and *n* = 7 in AS group.

Supplementary Figure 2 The representative images of embryos as a whole as followed in the Control-MO and *cingulin b*-MO groups.

Supplementary Table 1 Primers for the synthesis of objective genes in WISH experiment.

Supplementary Table 2 Primers for Real-Time PCR experiment.

## Abbreviations

DEGs: differently expressed genes
HCs: hair cells
hpf: hours post-fertilization
JNK: Jun N-terminal kinase
MAPK: Mitogen-activated protein kinase
PLL: posterior lateral line
qRT-PCR: quantitative real-time PCR
TJ: tight junctions
WISH: whole mount *in situ* hybridization
GFP: green fluorescent protein.
