# Supplementary material for: Cingulin b Is Required for Zebrafish Lateral Line Development Through Regulation of Mitogen-Activated Protein Kinase and Cellular Senescence Signaling Pathways
Source: Front Mol Neurosci. 2022 May 5;15:844668. doi: 10.3389/fnmol.2022.844668 (PMC9119177; doi:10.3389/fnmol.2022.844668)
Supplement: Supplementary Figure 1 — The representative S (sense control) and AS (antisense mRNA probe) images of cingulin b in the PLL of zebrafish at 48 hpf. Black arrowheads indicate neuromasts. Scale bars represent 50 μm, n = 10 in S group, and n = 7 in AS group. [file Data_Sheet_1.pdf]

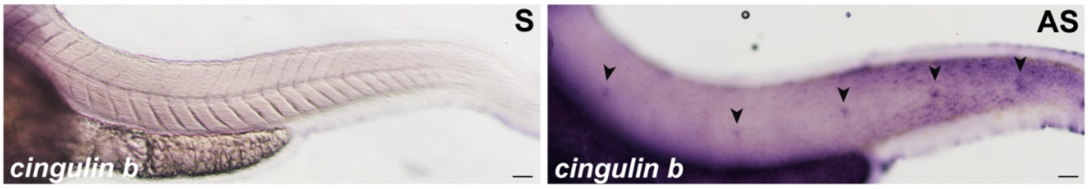

Supplementary Fig 1. The representative S (sense control) and AS (antisense mRNA probe) images of *cingulin b* in the PLL of zebrafish at 48 hpf. Black arrowheads indicate neuromasts. Scale bars represent 50 μm, n = 10 in S group, and n = 7 in AS group.

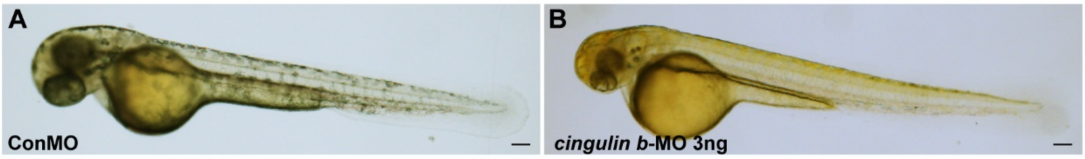

Supplementary Fig 2. The representative images of embryos as a whole as followed in the Control-MO and *cingulin b*-MO groups.

Table S1. Primers for the synthesis of objective genes in WISH experiment.

Gene	Application	Primers
cingulin b	WISH	5'-CAAGAGTCGACTGGCCAATG-3'
		5'-CCTGATCCTCATCCTGTGCA-3'
eya1	WISH	5'-TAGCACAGACGGATTCCACG-3'
		5'-TGGTGCTATCAGAACGCAGA-3'
atoh1a	WISH	5′-GTCAAAGTACGCGAGCTCTG-3′
		5′-ACTTCAGTGAGGCGAGAACT-3′

Table S2. Primers for Real-Time PCR experiment.

Gene	Application	Primers
gapdh	qPCR	5’-CCAACTGCCTGGCTCCTT-3’
		5’-CCCATCAACGGTCTTCTGTG-3’
arr3a	qPCR	5’-AAGACCTGGACGTGATTG-3’
		5’-TTGAAAGTGAAGGGATGG-3’
akt2	qPCR	5’-AACAGAGGCTTGGCGGAGGT-3’
		5’-TCCTCCGCGTCAAGACTGTCA-3’
arr3b	qPCR	5’-CTCTGACACAGAAACGTTAGCAG-3’
		5’-CACCATCAACTGAATCAACGC-3’
akt3b	qPCR	5’-TTCTCTGTCGCCAAGTGTCAGCT-3’
		5’-ACCCACTCGTCCCTCTCCTCT-3’
tp53	qPCR	5’-CAAACGTAGTTTGGTGAAAGAATC-3’
		5’-GCAGGCACCACATCACTTAAC-3’
gadd45aa	qPCR	5’-ATTGAAAGGATGGACTCGGTG-3’
		5’-TTTCAGTTCTGCTCATCGCTC-3’
mapk3	qPCR	5’-ATGGCGGAATCGGGCAGTAGCGCGGC-3’
		5’-TAGCAGATATGGTCRTTGCTGAG-3’
mapk1	qPCR	5’-CCTGCTCCGCTTCAAGCAT-3’
		5’-GTCTCCATCAGGTCCTGTACGATG-3’
hsp70.1	qPCR	5’-ACAAGCCATCAATACGCCTG-3’
		5’-GCCCAGGTCAATGCCAATAG-3’
mapk12b	qPCR	5’-AGCCATTGACCGCAAGACAG-3’
		5’-CATCCAGCAGCCCGATAACA-3’
mef2ca	qPCR	5’-TTGCGCGGATAATGGACGAACG-3’
		5’-GGGGGCCGGTGGGTGACTC -3’
cacna1fb	qPCR	5’-CAACGCCACAAACCATGACC-3’
		5’-AGCCGTT GCGGATGTATGAG-3’
ppp3r1a	qPCR	5’-GCCTTTAGGATCTACGACATGG-3’
		5’-ATATTCTCCCGTCTCCGTCTTT-3’
ppp3cca	qPCR	5’-CTGCTTAGACGACATTAGG-3’
		5’-CTGACGGAGTTGTGGTTA-3’
rac1l	qPCR	5’-TGGAAGCAGCGGCAGTAAATGC-3’
		5’-ATGTTTTTCCCACAGCCCCGTC-3’
rac3a	qPCR	5’-AAGACGTGCCTGCTCATCAGCT-3’
		5’-TCAGGGTACCACTTTGCCCTGA-3’
foxo3b	qPCR	5’-TCATCTTCAAGGAGGAATGC-3’
		5’-GGATGGAGTTCTTCCAACCA-3’
mapta	qPCR	5’-GCCCAATGCTCACTACAACTCC-3’

		5'-TGTCTCATCCCCTCTTGAATCCT-3'
zgc:114130	qPCR	5'-CCAGAAAGTATCTTCCCAACCACA-3'
		5'-TCCAGCGTTACCCTCCACCA-3'
lin37	qPCR	5'-TGATGCGGTGTTACAAGGTCTG-3'
		5'-GCGTGGTGGTGCTAAACTGAG-3'
atf7b	qPCR	5'-GCCGAGGCTGTAGCTGTTTTA-3'
		5'-TGGATGTGGGCTGTGATTGG-3'
cdkn1a	qPCR	5'-ATGCAGCTCCAGACAGATGA-3'
		5'-CGCAAACAGACCAACATCAC-3'
cingulin b	qPCR	5'-AGGACTCACAGAAAATCACCAA-3'
		5'-GAACAGCTCTTTCTTTACACGG-3
